# Systems Analysis of Human Visuo-Myoelectric Control Facilitated by Anodal Transcranial Direct Current Stimulation in Healthy Humans

**DOI:** 10.3389/fnins.2018.00278

**Published:** 2018-04-30

**Authors:** Vinh Kha, Aguida S. Foerster, Susan Bennett, Michael A. Nitsche, Filip Stefanovic, Anirban Dutta

**Affiliations:** ^1^Department of Biomedical Engineering, University at Buffalo, The State University of New York, Buffalo, NY, United States; ^2^IfADo Leibniz Research Centre for Working Environment and Human Factors (LG), Dortmund, Germany; ^3^Department of Rehabilitation Science, University at Buffalo, The State University of New York, Buffalo, NY, United States; ^4^Department of Neurology, University Medical Hospital Bergmannsheil, Bochum, Germany

**Keywords:** non-invasive brain stimulation, transcranial direct current stimulation, visuomotor task, response time, myoelectric control

## Abstract

Induction of neuroplasticity by transcranial direct current stimulation (tDCS) applied to the primary motor cortex facilitates motor learning of the upper extremities in healthy humans. The impact of tDCS on lower limb functions has not been studied extensively so far. In this study, we applied a system identification approach to investigate the impact of anodal transcranial direct current stimulation of the leg area of the motor cortex via the human visuo-myoelectric controller. The visuo-myoelectric reaching task (VMT) involves ballistic muscle contraction after a visual cue. We applied a black box approach using a linear ARX (Auto-regressive with eXogenous input) model for a visuomotor myoelectric reaching task. We found that a 20th order finite impulse response (FIR) model captured the TARGET (single input)—CURSOR (single output) dynamics during a VMT. The 20th order FIR model was investigated based on gain/phase margin analysis, which showed a significant (*p* < 0.01) effect of anodal tDCS on the gain margin of the VMT system. Also, response latency and the corticomuscular coherence (CMC) time delay were affected (*p* < 0.05) by anodal tDCS when compared to sham tDCS. Furthermore, gray box simulation results from a Simplified Spinal-Like Controller (SSLC) model demonstrated that the input-output function for motor evoked potentials (MEP) played an essential role in increasing muscle activation levels and response time improvement post-tDCS when compared to pre-tDCS baseline performance. This computational approach can be used to simulate the behavior of the neuromuscular controller during VMT to elucidate the effects of adjuvant treatment with tDCS.

## Introduction

**Figure 1:**
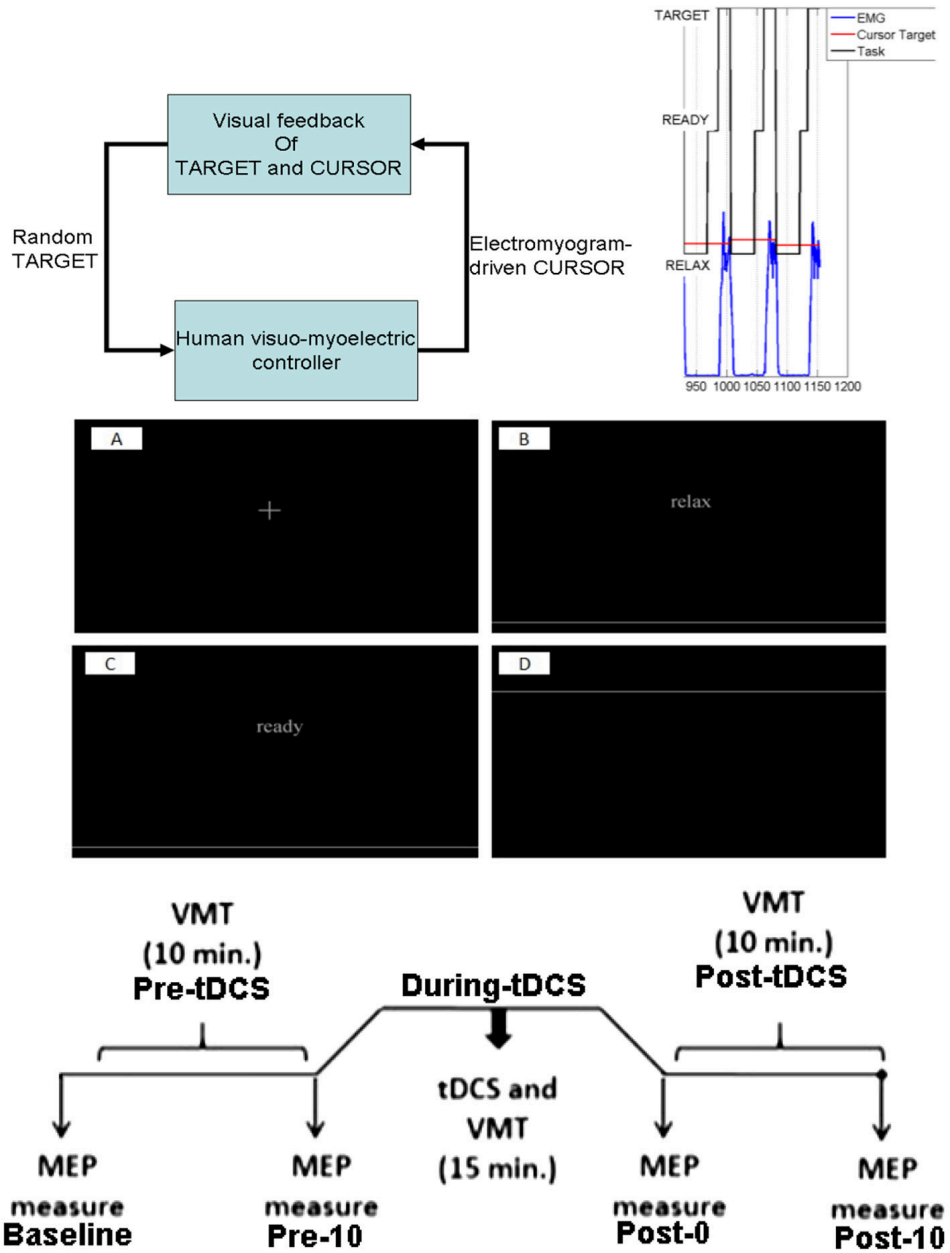
Experimental protocol. **Top**: for the reaching task, a random TARGET was shown on a computer monitor. The participants were instructed to contract their tibialis anterior muscle to reach that target. Each muscle contraction cue of 5 s duration was preceded by a RELAX time (10 s) and a READY time (3–5 s) during a trial, which was provided on the computer monitor. **Middle**: the computer monitor provided visual feedback of the task components, fixation (A), RELAX (B), READY (C), and TARGET presentation (D). **Bottom**: Protocol for tDCS during the visuomotor task and the TMS-MEP measures.

Non-invasive brain stimulation (NIBS) techniques, such as transcranial direct current stimulation (tDCS)—an electrically based intervention—can modulate brain activity to promote neuroplasticity (Nitsche and Paulus, 2000). Following initial studies in healthy humans (Nitsche and Paulus, 2000), numerous subsequent tDCS studies have been performed by various research groups around the world that showed that tDCS can alter motor cortex excitability, and performance of the upper extremities. In one of the first studies on application of tDCS for the leg area of the motor cortex, Jeffery and colleagues (Jeffery et al., 2007) showed that anodal tDCS transiently enhanced the excitability of the contralateral leg motor cortex in healthy subjects. Tanaka et al. (2009) showed that tDCS could improve leg motor function in healthy humans. Excitability-enhancing anodal tDCS transiently enhanced maximal leg pinch-force, but not reaction time during its application. Roche et al. reported short-term modulatory effects of anodal tDCS over the leg M1 on the excitability of lumbar spinal circuits (Roche et al., 2011) and lumbar propriospinal system excitability (Roche et al., 2012). The authors concluded that anodal tDCS increased general excitability of the stimulated cortical area, and that respective excitability changes activated descending controls involved in co-contraction. Our preliminary results from healthy humans in a myoelectric target reaching task (see Figure 1) that involved quick initiation/termination of muscle activation (Dutta et al., 2014; Foerster et al., 2015; Kumar et al., 2016) showed that offline anodal tDCS over the leg M1 (Dutta et al., 2014) decreased the delay in the initiation of isometric contraction of the tibialis anterior (TA) muscle, when compared to sham tDCS. A limited number of studies in humans on tDCS effects over the leg M1 have revealed neuroplastic changes at multiple levels of cortical and spinal networks, which affect “direct control” (Tanaka et al., 2009; Madhavan et al., 2011) as well as “gating” actions of motor cortex output neurons on spinal circuits (Roche et al., 2009, 2011, 2012).

**Figure 2:**
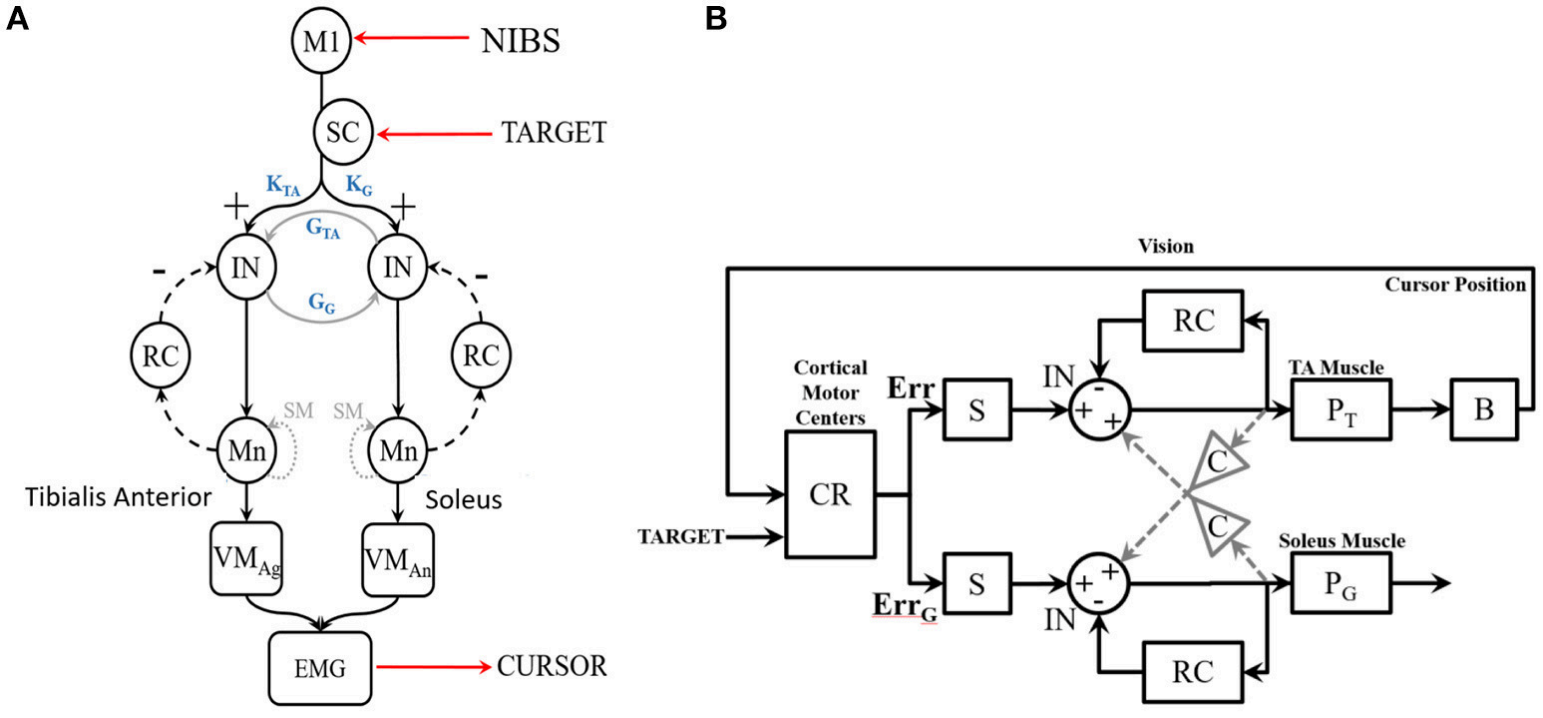
**(A)** Diagram of the SSLC at the spinal level including: Interneurons (IN) that connect afferent and efferent divisions of neural projections and integrate sensory information; Renshaw Cells (RC) that provide copies/approximations of efferent neural signals to predict system dynamics or consequences; Motoneurons (Mn) that drive activation of the muscle fibers; Superior Colliculus (SC) that projects a visually derived gain field due to effector-target error; and Spindles (SM) that act as muscle sensors to deliver muscle stretch length and velocity information to the CNS. The topology also includes a Virtual Muscle (VM) agonist (VMag) and antagonist (VMan). **(B)** The SSLC adopted for the isometric visuo-myoelectric reaction time task (VMT). SSLC is comprised of the following components: R, cortical commands issued to the SSLC in the form of a sigmoidal activation function; D, signal delays between cortical and spinal circuits; G, collicular gain fields projected to spinal circuits; M, Renshaw feedback of motoneuron activity; P, virtual muscle models; C, a scaling factor of co-contraction between the two sides in the controller. Also, the following signals: e, motor drive signals projected to the spinal-like circuits; U, the motor activation level for each muscle model; Y, the generated muscle tension. Subscripts T and G denote the agonist and antagonist muscles, respectively.

Preliminary results showing cortical (Tanaka et al., 2009; Madhavan et al., 2011) as well as spinal effects (Roche et al., 2009, 2011, 2012) of anodal tDCS motivated our investigation of the short-term modulatory effects of tDCS on cortical vs. spinal parts of the central nervous system by simulating the effects using a tunable spinal-like controller (Stefanovic and Galiana, 2014a) that represents behavioral patterns during a lower limb reaching task (Stefanovic and Galiana, 2014a). These studies have shown that the slope of the input-output (I-O) curve of motor evoked potentials (MEP) increases after anodal tDCS over M1, which is an index of enhanced cortical excitability (Nitsche et al., 2005). Increased cortical excitability is postulated to affect systems performance during the visuomotor task (Foerster et al., 2015), which involves discrete cued reaching movements of the cursor. In accordance to our postulate, the computational model directly connecting primary motor cortex (M1) to spinal circuits (see Figure 2) is based on the results from transcranial magnetic stimulation (TMS) of the human nervous system which showed that corticospinal neurons make powerful and direct connections with most alpha motoneurons in the spinal cord, and these connections likely control for muscle contractions (Mills, 2000). These corticospinal projections have however also inhibitory effects on the spinal cord via spinal interneurons. These can “gate” the spinal circuits that receive peripheral inputs, and therefore enable quick responses to ongoing changes required for adaptation of complex behaviors. Although functional effects on motor performance are obtained, mechanisms are not clarified during a myoelectric target reaching task (see Figure 1) which could include cortical and spinal components. Here, delineation of cortical vs. spinal effects of anodal tDCS might then be crucial for optimization of respective interventions aimed to improve motor performance. Although the primary motor cortex can be targeted using Finite Element Method (FEM) models based on high-resolution magnetic resonance imaging (MRI) scans that can identify the electric field at the targeted neural tissue in the brain (Truong et al., 2015), however, the physiological and behavioral effects of the electric field cannot be predicted by FEM alone. Therefore, mathematical representations of the respective dynamic system based on experimental data from behavioral tasks may help to delineate cortical vs. spinal effects of anodal tDCS. Moreover, systems analysis can be applied to understand subject-specific effects, which is relevant because tDCS does not produce identical treatment effects in each individual (inter-subject variability) (Madhavan et al., 2016).

## Methods

In this study, system-identification techniques were applied to characterize visuomotor control. During the visuomotor task (VMT), the EMG of the TA muscle, as shown in Figure 1, controls the cursor. There were three VMT sessions, one is the Pre-tDCS, next is during-tDCS, and the final session is Post-tDCS. The visual feedback of the cursor, as well as the target, are provided to the subject. System identification, the construction of mathematical models of dynamic systems from input/output measurements, has been extensively used to characterize visuomotor tasks (Baddeley et al., 2003), which is also the focus of the current study. Specifically, we propose an open-loop transfer function (i.e., open-loop identification), which can be represented by a high-order finite impulse response (FIR) filter in the time domain, where the feedback contribution to the input spectrum is considered to be negligible. Identification of the FIR filter requires no knowledge of the feedback in the system and very few apriori assumptions, i.e., the span of the memory of the system. A poor noise model can introduce a bias into the model, which was investigated based on the residual analysis. Specifically, the bias will be small when the feedback contribution to the input spectrum is small, however after the fast initial myoelectric control of the cursor to reach the target, maintaining the cursor at the target requires visual feedback, which may substantially affect the input spectrum in the model. In that case, spectral analysis of the closed-loop system using the open-loop transfer function may not provide correct results, and we need to incorporate the feedback, e.g., using gray-box modeling.

We first leveraged a linear black-box model, the linear Auto-regressive with eXogenous input (ARX) model, which is the simplest input and output polynomial model that can be fitted using the System Identification toolbox of Matlab (Mathworks Inc., USA). In the ARX model, the parameters of the error term are linear, and the linear least squares method can be used for parameter estimation. This method is numerically simple and reliable and avoids problems of local minimum and convergence. This ARX model can be used to investigate the transfer function using its gain/phase margin. Then, for gray-box modeling (see Figure 2), we postulate that simulations of tDCS effects on a myoelectric controller (Dutta et al., 2014) that adopts a spinal-like network topology for a reaching task (Stefanovic and Galiana, 2014a) can be used to assess the effects of tDCS over the leg M1 representation. Here, the Simplified Spinal-Like Controller (SSLC) model is based on known physiological characteristics of the spinal circuitry (Stefanovic and Galiana, 2014a,b, 2016), including: Interneurons (IN) that connect afferent and efferent divisions of neural projections and integrate sensory information, Renshaw Cells (RC) that use efference feedback to predict system dynamics or its effects, Motoneurons (Mn) that drive the stimuli to the muscle fibers, and Spindles (SM) that act as muscle sensors to deliver muscle stretch length and velocity information to the CNS.

**Figure 3:**
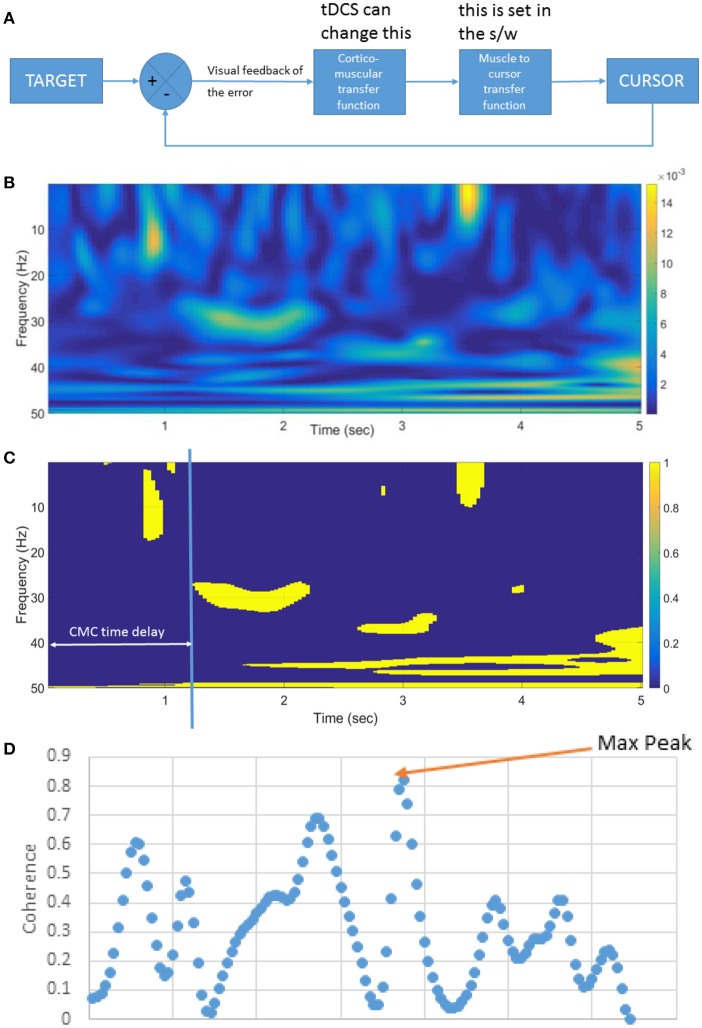
**(A)** Block diagram of the linear time invariant VMT system. tDCS is postulated to affect the cortico-muscular transfer function. The muscle to the cursor mapping is set in the software (s/w). **(B)** An illustrative example of the pre-tDCS cortico-muscular transfer function shown by corticomuscular coherence (CMC) during muscle contraction (in VMT). **(C)** Significance of the CMC, and CMC time delay, **(D)** Beta-range (13–30 Hz) CMC peak during static EMG output required during the muscle contraction task.

In this study, the target input and the cursor output define the open-loop VMT system, while the EEG input and EMG output represent the plant model for the cortico-muscular transfer function (see Figure 3A). Corticomuscular coherence (CMC) is a measure of the relationship between electroencephalogram (EEG) and EMG (Salenius et al., 1997; Witham et al., 2011) during muscle contraction, and a physiological parameter involved in task performance. In a lower-limb study on EEG-EMG beta-band coherence (Gwin and Ferris, 2012), a significant coherence between electrocortical signals of the contralateral motor cortex and EMG in the beta-range (13–30 Hz) and gamma-range (31–45 Hz) was found for different exercise types, e.g., isometric and isotonic, knee, and ankle exercises. The study showed furthermore that gamma-range coherence was significantly larger for isotonic exercises, while isometric contractions favor beta-range oscillations. It has been demonstrated that CMC originates from primary motor cortex (M1) (Gerloff et al., 2006), and therefore tDCS of M1 affecting cortical excitability is postulated to affect CMC (Power et al., 2006). Therefore, we also investigated the significance of the CMC and CMC time delay during VMT.

### Subjects

Ten healthy right leg-dominant male (4) and female (6) volunteers (age: 26 ± 5 years) participated in this study after giving informed consent, and all experiments were conducted in accordance with the Declaration of Helsinki. The study obtained ethics approval at the University Medical Center, Goettingen, Germany. Subjects who (i) were taking any acute or regular CNS-active medication, and (ii) had a history of neurological, psychiatric, or medical disease, family history of epilepsy, pregnancy, cardiac pacemaker or previous surgery involving metallic implants at the time of the study, were excluded from participation.

### Visuo-myoelectric control task

The brain-computer interface (BCI) for VMT, as shown in Figure 1, is presented in detail in our earlier publication (Foerster et al., 2015). The subject needs to make a muscle contraction as fast as possible to reach the target and then maintain the cursor as close to the target as possible. Each muscle contraction cue of 5 s duration was preceded by RELAX time (10 s) and READY time (3–5 s) during a VMT trial, presented by Psychtoolbox (http://psychtoolbox.org/) GUI—see bottom panel of Figure 1. Fifteen VMT trials were conducted before and after 15 min of anodal tDCS over M1. Anodal tDCS (StarStim, Neuroelectrics, Spain) with 2 mA was conducted for 15 min with the anode (5 × 7 cm) positioned at Cz (international 10–20 system of scalp sites) and the cathode (5 × 7 cm) placed over the right supraorbital ridge at FP2 (international 10–20 system of scalp sites). The electromyogram (EMG) was collected from the tibialis anterior (TA) muscle of the right (dominant) leg, amplified and band-pass filtered (anti-aliasing, frequency band = 10–500 Hz) before being sampled at 2,000 Hz by a 12-bit data acquisition (DAQ) system (NI USB-6009, National Instruments, USA). The EMG was recorded on a PC by Signal software (http://www.ced.co.uk/pru.shtml?sig3wglu.htm) via a 16-bit DAQ for offline analysis. Cortico-spinal excitability was monitored before and after tDCS with TMS (Groppa et al., 2012). TMS was used to identify the motor cortical localization (hotspot) of the right TA muscle. The hotspot was determined as the TMS coil position resulting in the largest motor evoked potential (MEP) of the target muscle (Groppa et al., 2012), elicited by a Magstim 200 stimulator (Magstim, Dyfed, UK) with a figure-of-eight coil (diameter 70 mm). Eyes-open resting-state EEG was recorded at 500 Hz from the central site Cz (international 10–20) as well as the nearby electrodes F3, F4, P3, P4 (international 10–20 system) before and after anodal tDCS over Cz. During tDCS, the EEG could not be recorded from Cz, but only from the nearby electrodes F3, F4, P3, P4 (international 10–20 system), which were interpolated with spherical splines (Scherg et al., 2002) to estimate EEG at Cz (virtual electrode) using the EEGLAB “eeg_interp()” function (Delorme and Makeig, 2004). For task performance, the participants had to contract the TA muscle isometrically as fast as possible in response to a visual cue, the TARGET. In each trial, the TARGET jumped to a randomized value between 40 and 80% of maximum voluntary contraction (MVC), i.e., random TARGET. Myoelectric visual biofeedback was presented with proportional system dynamics, and the participants were instructed to adjust EMG activity to best match the CURSOR to the TARGET level. The average rectified EMG during 3 s of MVC was used for normalization of the EMG during VMT. The moving average of the normalized rectified EMG from the TA muscle was provided as CURSOR along with the TARGET signal. The participants already learned the VMT in a practice session before performing the experiment. The VMT sessions contained 13 trials and a total duration is roughly 10 min, as shown in the bottom panel of Figure 1. We analyzed the MVC-adjusted EMG-driven random TARGET pursuit during VMT performance to estimate the model for the human visuo-myoelectric controller as well as to calculate EMG response latency, CURSOR response latency, and response accuracy—see top panel of Figure 1.

### ARX black-box model fitted to TARGET (input)-CURSOR (output) VMT data

Our goal was to perform a system identification using the FIR model and its gain/phase margin analysis to find out how performance of the human myoelectric controller changed during visuomotor task performance due to tDCS (comparing Pre-tDCS and Post-tDCS VMT sessions). We first investigated the human visuo-myoelectric controller using a black box system identification approach, specifically a single-input single-output polynomial model (“polyest” in Matlab). Trial-to-trial variation was investigated to understand how the brain optimized response time for the initially ballistic (open-loop) task component, and response accuracy moderated by visual feedback during the myoelectric control task component. We fitted a FIR model between the TARGET and the CURSOR. A respective block diagram for VMT is shown in Figure 3A. We used the system identification app of Matlab (The Mathworks, Inc. USA) for selecting model order based on the Akaike Information Criterion, and estimated the FIR model parameters using the least squares method. We identified the best order for the ARX model based on pre-tDCS experimental data and then fitted the model to each trial pre and post-tDCS. We then used the fitted ARX model to identify gain/phase margins pre and post-tDCS to identify tDCS-caused changes from baseline values. We hypothesized that there would be a movement of the zeroes of the ARX model (poles are all at origin—FIR model) during VMT due to tDCS intervention. The highest model order of the regularized ARX that suited all subjects was present in the pre-tDCS TARGET (input)-CURSOR (output) data based on the Akaike information Criterion (Akaike, 1974). Since it has been demonstrated that CMC originates from M1 (Gerloff et al., 2006), it was postulated that anodal tDCS of M1 will affect CMC (Power et al., 2006). CMC between the EEG at Cz and EMG was calculated using Wavelet cross-spectrum analysis (WCS). A new statistical test developed by Bigot and colleagues (Bigot et al., 2011) was conducted to find significant CMC values based on WCS. Then, the maximum CMC value during the muscle contraction (task D during VMT—see the middle panel of Figure 1) was defined as the CMC peak, as shown in the Figure 3D. The CMC time delay was defined by the delay in reaching the significant value (Bigot et al., 2011) after the appearance of the visual cue, as shown in Figure 3B. Since VMT involved isometric contractions, CMC was highest in the beta-range during muscle contraction (5 s), as shown in Figure 3C. We postulated that tDCS would change the corticomuscular transfer function (see Figure 3A), which will be reflected in the CMC peak (Figure 3D) and CMC time delay (Figure 3C). A custom Matlab script was written for these computations.

### SSLC gray box model simulation using input-output function of motor evoked potentials

We simulated the human visuo-myoelectric controller (Dutta et al., 2014) with a novel gray box model that adopts a spinal-like network topology (Stefanovic and Galiana, 2014a), as shown in Figure 2B. The SSLC was adapted from our earlier studies (Stefanovic and Galiana, 2014a,b, 2016) to simulate the VMT. The SSLC is a model of the neural topologies that drive neuromuscular control and coordination at the spinal level. We first postulated that anodal tDCS effects on M1 will affect the descending behavior of the SSLC in the s-domain (Laplace transformation) without visual feedback, i.e., open-loop transfer function—see Figure 2B:

$$\begin{array}{l} Y_{T}\left(s\right)=U_{T}\left(s\right)P_{T}\left(s\right)\\ U_{T}\left(s\right)=G_{T}e_{T}\left(s\right)-U_{T}\left(s\right)M_{T}\left(s\right)+CU_{G}\left(s\right) \end{array}$$

Then, by rearranging the equation:

$$\begin{array}{l} U_{T}\left(s\right)=\frac{G_{T}e_{T}\left(s\right)+CU_{G}\left(s\right)}{1+M_{T}\left(s\right)} \end{array}$$

In addition, *e*_*T*_(*s*) is defined by:

$$\begin{array}{l} e_{T}\left(s\right)=R\left(s\right)D\left(s\right) \end{array}$$

Where *D* is the time delay from the cortical centers to the spinal centers, *f* (*t*– δ), and whose Laplace Transform is represented by,*e*^−δ*s*^. Thus, for the tibialis anterior muscle,

$$\begin{array}{l} e_{T}\left(s\right)=R\left(s\right)e^{-\delta s}\\ U_{T}\left(s\right)=\frac{\underset{Cortical\;Command}{\underset{︸}{e^{-\delta s}G_{T}R\left(s\right)}}+\underset{Co-contraction}{\underset{︸}{CU_{G}\left(s\right)}}}{1+M_{T}\left(s\right)} \end{array}$$

Similarly for the gastrocnemius muscle,

$$\begin{array}{l} U_{G}\left(s\right)=\frac{e^{-\delta s}G_{G}R\left(s\right)+CU_{T}\left(s\right)}{1+M_{G}\left(s\right)} \end{array}$$

According to these equations, the motor activation level is directly proportional to two components, the cortical command, R(s), and the co-contraction.

We hypothesized that anodal tDCS changes the gain of corticomuscular control by changing the input-output (I-O) function of the primary pyramidal neurons (Lafon et al., 2017), that constitutes the descending cortical command. Anodal M1 tDCS increases cortical excitability of M1. Theoretical analysis reveals that excitatory synaptic strength controls the threshold of the neuronal input-output (I-O) function, while inhibitory synaptic strength alters the threshold and gain (Carvalho and Buonomano, 2009). This assumption is supported by the sigmoidal curve characteristics of the TMS-motor evoked potential (MEP) stimulus–response (SR) (Klomjai et al., 2015). Here, it was assumed that a single pulse of TMS produces excitation of the corticospinal neurons in the primary motor cortex. Therefore, it is postulated based on the SSLC that a decrease of cortical excitability (e.g., due to cathodal M1 tDCS) will reduce co-contraction while cortical excitability enhancing anodal M1 tDCS will increase co-contraction (as shown experimentally by Roche et al., 2011). Particularly, the IN combines signals from RC, SM, and antagonist INs in an excitatory (+) and inhibitory (-) summation based on the drive of the transmitting neuron (node). Also, the IN cross-connections between opposite/symmetric components are scaled by a gain, G, so that the interactions between them (e.g., antagonist muscle groups) can vary (Stefanovic and Galiana, 2014a). The SM provides muscle stretch sensory information of the muscle model in the form of musculotendon stretch length (MT) and stretch-length velocity (MTv). The RC provide copies of efferent Mn activity to the IN (Stefanovic and Galiana, 2014a, 2016). The neuromuscular output is thus defined by the length-tension-stimulus relationship of the muscle at a given instant in the form of a predictive model (i.e., learned muscle response; Cheng et al., 2000; Raphael et al., 2010), or as approximation of the predicted SM output (e.g., MT or MTv; Lan and He, 2012). Here, subject-specific TMS-MEP SR curves capturing cortical excitability alterations were modeled by the input-output (I/O) curve (Möller et al., 2009). The MEP response measured with TMS represented transsynaptic activation of the pyramidal cells evoking descending volleys in the pyramidal axons evoking a motor-evoked potential (MEP) on EMG of the target muscle (Klomjai et al., 2015).

For fitting baseline (pre-tDCS) VMT data in a closed-loop condition, the SSLC will need visual feedback (Stefanovic and Galiana, 2014a,b, 2016), as shown in Figure 2B. Here, visual task-error signals drive motor commands to the spinal-like centers, which in turn coordinate and execute motor neuron activity. The projected cortical commands manifest as a sigmoid function (called “motor urgency” Thobois et al., 2007) that scales controller behavior (block S in Figure 2B). This sigmoid function was determined by the TMS SR curve, that is widely used to assess motor cortex excitability (Peri et al., 2016). Err is the error drive (difference between cursor position from the target) computed in block CR (in Figure 2B) that includes the cerebello-thalamo-cortical circuit (Ide and Li, 2011). Also, cortical and midbrain collicular areas project gain-fields that can scale a movement temporally or spatially. The combined motor drive is sent to the muscles that generate tension and movement. Each component of the SSLC is organized as a block (see Figure 2B) within a Simulink (Mathworks Inc., USA) framework based on its functionality and neural topology as in earlier studies (Stefanovic and Galiana, 2014a, 2016). Subject-specific pre-tDCS and a post-tDCS Boltzmann sigmoidal functions experimentally found by standard TMS methods (Kukke et al., 2014) will define block S of the SSLC, as shown in Figure 2B. The controller has been used in our previous studies to demonstrate contributions of the spinal-like motor centers to movement execution and co-ordination in complex tasks, as well as dynamics between cortical and spinal-level motor control. As such, the SSLC can be used as a tool to model changes in motor excitability due to tDCS and how the effects manifest in the cortical-spinal-muscular controller.

In this particular study, we were interested in applying the SSLC to control the lower limb in an isometric task. First, the muscle model for the SSLC was developed using Virtual Muscle 4.0.1 (USC, USA) (Cheng et al., 2000), and muscle morphometry data from the anterior tibial muscle (Helliwell et al., 1987). The respective Matlab program generates a Simulink block that emulates behavior and dynamics of muscles. Following this, the biomechanics of the system was changed to reflect the isometric experiment. Since isometric tasks do not include any motion, the task is defined as relation between TA contraction (i.e., EMG) and CURSOR position. Using the TA EMG data from the experiment, and the causal CURSOR movement, a respective fit was generated in Matlab (Mathworks Inc., USA). The TA contraction to CURSOR relation is approximated by a 0 gain low-pass filter:

$$\begin{array}{l} B=\frac{1}{1+\frac{s}{2\pi 60}} \end{array}$$

The remaining block components of the SSLC remain unchanged as shown in Figure 2. However, as described, projected cortical commands manifest as a sigmoid function (called “motor urgency” Thobois et al., 2007) that scales controller behavior (Block S). This sigmoid function was determined by TMS-MEP SR curves, that are widely used to assess motor cortex excitability by TMS (Peri et al., 2016). We used two sigmoids, a pre-tDCS and a post-tDCS sigmoid identified by standard methods (Kukke et al., 2014) and fitted these to a Boltzmann sigmoidal function:

$$\begin{array}{l} \text{S}=\frac{{\text{P}}_{1}+\frac{{\text{P}}_{2}-{\text{P}}_{1}}{{\text{P}}_{2}}}{1+{\text{e}}^{-{\text{P}}_{4}\left(\text{Err}-{\text{P}}_{3}/10\right)}}+{\text{P}}_{1}/100 \end{array}$$

Err is the error drive defined by the cortical commands (difference of CURSOR position to TARGET distance in block CR). Respective *P*-values are found in the following table:

**Table:** 

	S_pre−tDCS_	S_post−tDCS_
P1	1.281	1.876
P2	4.606	5.207
P3	118.552	119.620
P4	0.117	0.122

Within the SSLC Simulink model, TARGET data are used as the input, while the cursor position is computed from the virtual muscle output and equation B. The cursor position is provided as a feedback to the cortical block, where it is compared to the TARGET, and the Err is calculated for the next iteration. The SSLC moves to the TARGET until it is reset to the zero position, in which case Err becomes zero. The process re-starts when the TARGET is presented again at a non-zero position. The algorithm changes the gains in steps over multiple iterations/loops and monitors the difference between the expected value (i.e., measurement data) and the real value (i.e., model output). Based on the direction of the calculated error of the two signals, the gains change iteratively until the error no longer changes, or is within a user-defined tolerance—typically within 5%. Therefore, the SSLC model fits to subject-specific pre-tDCS TMS-MEP SR curve and VMT data, which will be able to predict post-tDCS VMT data based on the post-tDCS TMS-MEP SR curve.

### Statistical analysis

EMG response latency was determined offline as the period from the instant of the visual TARGET cue to the moment when the rectified EMG in a sliding window of 500 ms from the TA muscle was more than three times of the standard deviation of the resting value (before presentation of the TARGET cue, in the READY state). The CURSOR response latency was determined offline as the duration from the instant the visual TARGET cue was provided for ballistic muscle contraction to the time the CURSOR crossed the TARGET during VMT. CURSOR response accuracy was computed as Root Mean Square Error (RMSE) between the CURSOR and the TARGET signals during the 5 s of TARGET cue presentation. We investigated post tDCS differences in EMG response latency, RMSE, CURSOR response latency, CMC peak, and CMC time delay from pre-tDCS baseline values using a two-way (trial X tDCS/Sham) ANOVA. In addition, post-tDCS changes in gain/phase margins from pre-tDCS baseline values were identified by a two-way (trial X tDCS/Sham) ANOVA. For *post-hoc* tests, Student's *t*-tests were applied.

## Results

**Figure 4:**
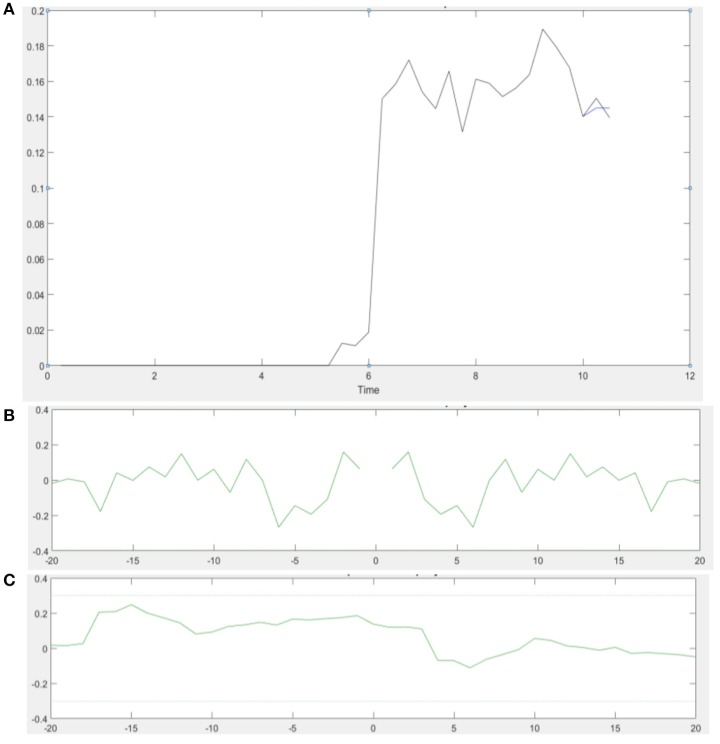
**(A)** An illustrative example of the model output of the 20th order FIR model in a single VMT trial. Muscle contraction takes place from 6 to 11 s (Time axis in sec). **(B)** Autocorrelation of the residuals plot shows that the residual autocorrelation function is in the range between the confidence intervals (dashed line) for uncorrelated signals—whiteness test. **(C)** Cross-correlation of the residuals with the model input also falls within the confidence intervals for uncorrelated signal—independence test.

**Figure 5:**
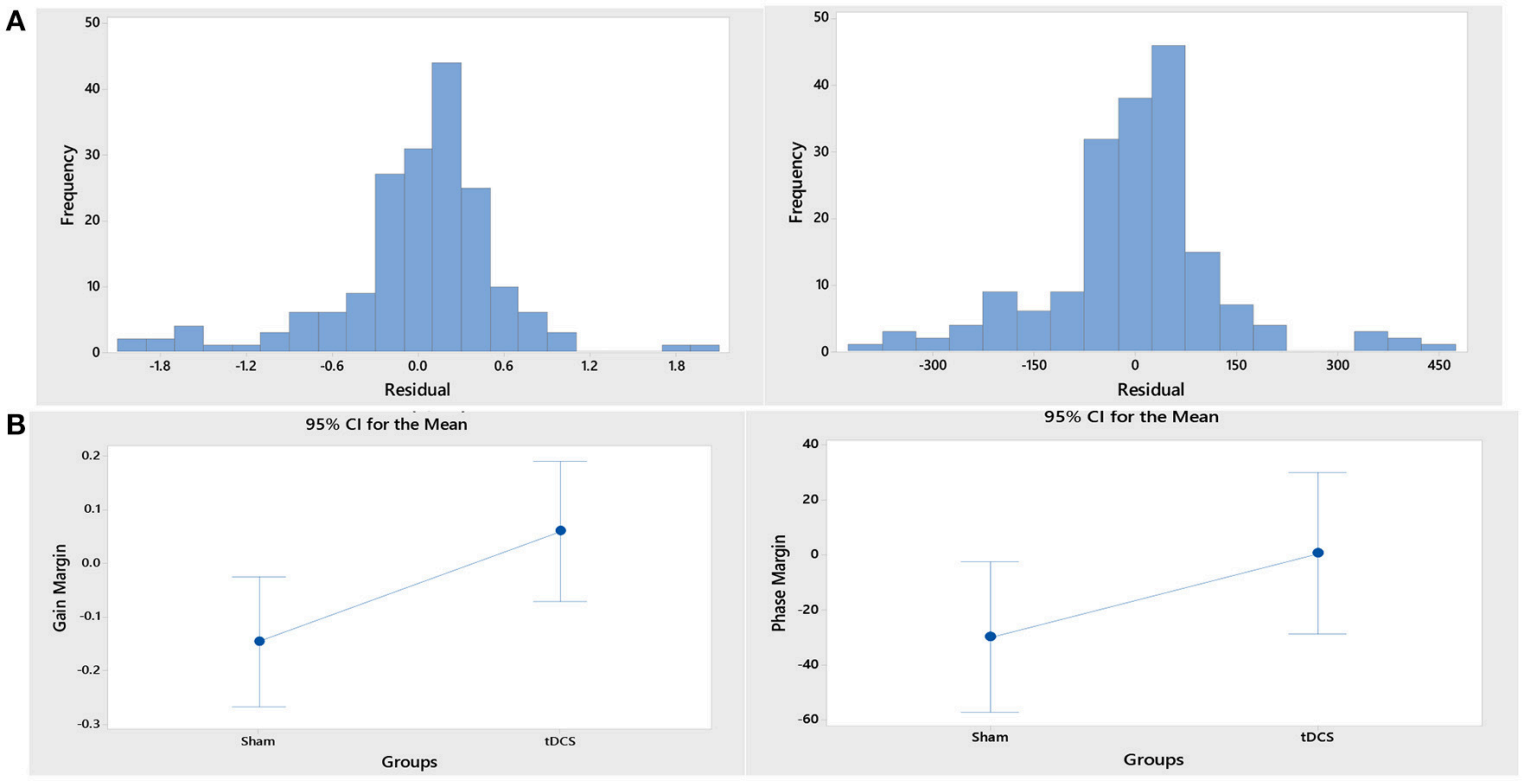
**(A)** Histograms of the residual of the ANOVA model for the response variables, “gain margin” (left panel) and “phase margin” (right panel). **(B)** Gain margin (left panel) as well as the phase margin (right panel) increased after anodal tDCS when compared to sham tDCS. The increase was significant for gain margin (*p* < 0.05) however not significant for phase margin (*p* = 0.143).

ARX models using least squares with regularization were estimated with the help of the system identification toolbox in Matlab (Mathworks Inc., USA). The 20th order FIR model was found to be suitable across subjects based on the AIC criterion. Figure 4A shows an illustrative example. The blue line represents the FIR model output fits (explained variance 98.65%), the black line represents the experimental CURSOR data with the TARGET serving as a step input. The autocorrelation of model residuals shown in Figure 4B is within the confidence interval (dashed line) for an uncorrelated signal, which shows that the residuals pass the whiteness test. Also, the cross-correlation of the model residuals with the model input shown in Figure 4C is within the confidence interval (dashed line) for an uncorrelated signal, which shows that the residuals pass the independence test. The estimated ARX system model was analyzed based on gain/phase margin. The two-way ANOVA results (see Figure 5) show that the factor intervention group (tDCS/sham) had a significant effect on the gain margin (*p* < 0.05), however not on the phase margin (*p* = 0.143). The trial effects and the interaction between trial and group were not significant for gain margin (0.649 and 0.781, respectively) as well as phase margin (0.435 and 0.865, respectively). Also, gain margin (left panel of Figure 5B) as well as the phase margin (right panel of Figure 5B) increased after anodal tDCS when compared to sham tDCS. The increase was significant for gain margin (*p* < 0.05) however not significant for phase margin (*p* = 0.143). In addition, there was a significant main effect of group (tDCS/sham) on the EMG response latency, CURSOR response latency as well as CMC time delay (*p* < 0.05). However, the main effect of the factor group (tDCS/sham) on the RMSE and the beta-range CMC peak was not significant.

**Figure 6:**
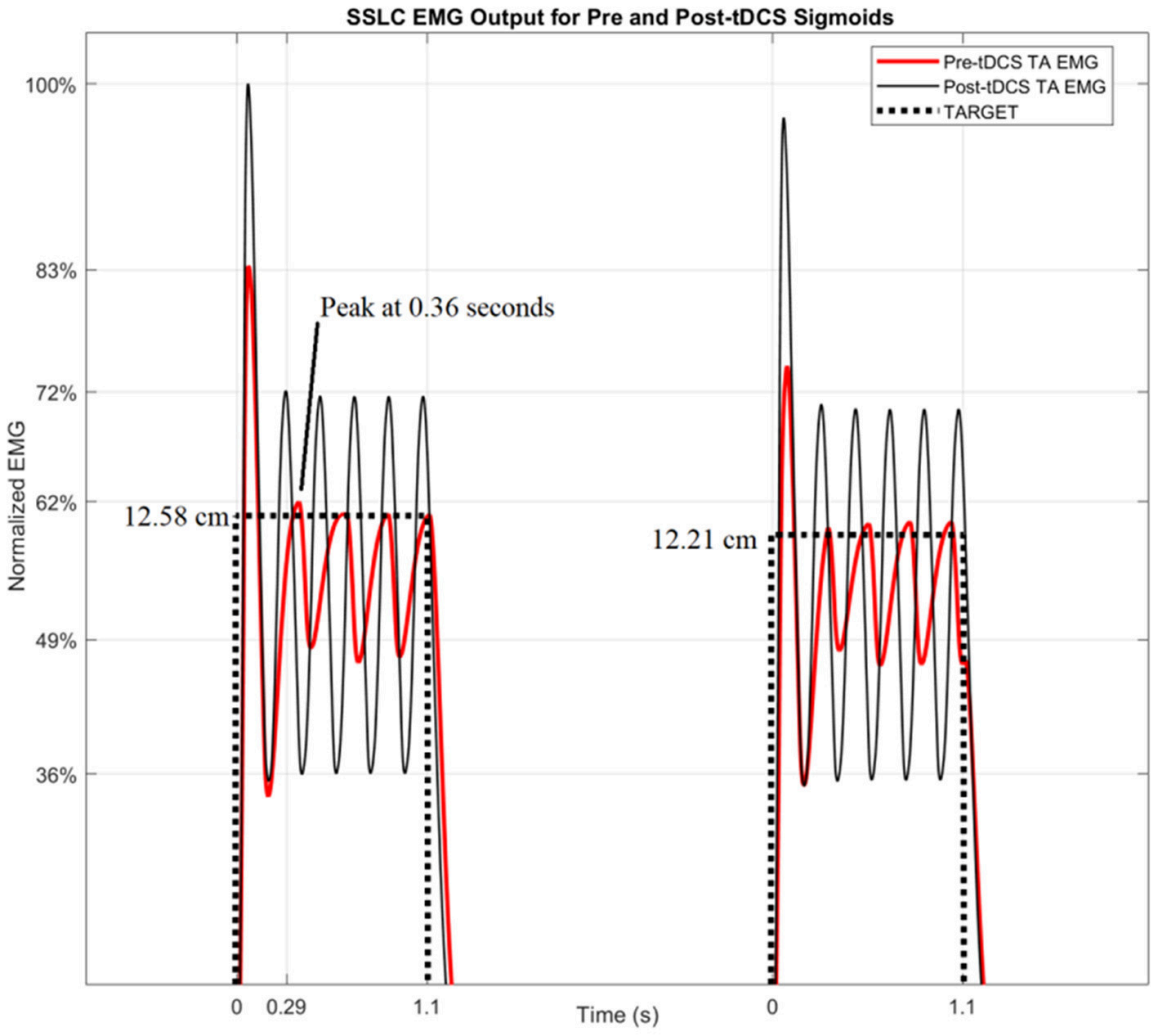
SSLC EMG Output for Pre and Post-tDCS Sigmoids. The SSLC model output is shown when performing the task to target (dotted line), as well as the EMG output when reaching to target using the pre-tDCS sigmoid (red line) and post-tDCS sigmoid (black line).

Figure 6 shows the TARGET reaching modeled by the SSLC, with TARGET input (dotted line), pre-tDCS TA EMG (red line), and post-tDCS TA EMG (black line). Since pre-tDCS and post-tDCS models are identical except for their sigmoidal functions that scale controller behavior (Block S), any variance in model outputs would be dependent on the cortical effects of tDCS captured by the I-O input functions. Figure 6 demonstrates that based on these changes, post-tDCS SSLC behavior shows a 17% higher normalized EMG output of the TA muscle when moving to the same target in relation to pre-tDCS SSLC behavior. Also, the time offset due to the dynamics in the myoelectric visual task shows a 70 ms faster response in the SSLC based on the first peak-times post-tDCS when compared to pre-tDCS. In addition, simulated TA EMG output shows a 10% increase of oscillations relative to the TARGET position post-tDCS when compared to pre-tDCS response.

## Discussion

In this study, we demonstrated the adequacy of a black-box system identification to capture ballistic myoelectric control for modeling of lower-limb VMT performance in healthy subjects. For the black-box modeling, the 20th order FIR modeled the TARGET-CURSOR dynamics well; the residuals satisfied the whiteness and independence tests. Satisfying the whiteness test shows that the residuals are uncorrelated, and the independence test shows that the residuals are uncorrelated with past inputs. For determination of the impact of anodal tDCS on task performance, the 20th order FIR model was investigated based on gain/phase margin analysis. The systems analysis showed a significant effect of anodal tDCS on the gain margin of the VMT system. After anodal tDCS, a significant (*p* < 0.05) increase in gain margin was found when compared to sham tDCS (see Figure 5B), which can be attributed to the controller behavior (Block S) in Figure 2B. In our related computational modeling work (Dutta and Nitsche, 2013), it was suggested that anodal tDCS modulates primarily the synaptic impulse response function (sIRF) of the dendritic tree of excitatory pyramidal neurons (ePN) in a cortical neural mass model (NMM). tDCS-induced changes of the synaptically-driven I-O function of ePN are modeled by the sigmoid function that scales controller behavior (Block S in Figure 2B) in SSLC (Stefanovic and Galiana, 2014a). Therefore, the sigmoid function was fitted to the TMS-MEP SR curve for gray-box modeling. Gray-box modeling results demonstrated that the post-tDCS increase in the slope of the sigmoidal function in block S of SSLC (see Figure 2B) due to anodal M1 tDCS can increase muscle activation/force levels (shown experimentally by Tanaka et al., 2009), and improve response time of task performance—see Figure 6. Particularly, based only on anodal M1 tDCS effects on the sigmoidal function of the I/O curve (i.e., an increase of its slope; Lafon et al., 2017), predicted SSLC response times improved by 70 ms and peak muscle output increased by 17%. Therefore, modeling of an isolated cortical effect of tDCS at the M1 produced a good qualitative fit to the experimental data.

Based on the computational modeling, we hypothesized that anodal tDCS enhances the responsiveness of ePN to afferent feedback (vision being one of them—see Figure 2B) in a non-specific way and increases the coherence between thalamus, cortex, and muscle. In our prior work (Dutta et al., 2015), we have found that surrogate measures of motor unit synchronization (identified using recurrence quantification analysis of surface EMG) were significantly higher in the tDCS group than in the control group. Also, Power and colleagues have demonstrated that increases in MEP size resulting from anodal tDCS of the motor cortex were paralleled by increases of intermuscular coherence in the EEG beta band (Power et al., 2006). Here, it can be speculated that increased synchronization (possibly reflected in the coherence measure) following anodal tDCS provides better binding of the sensorimotor elements leading to gain margin increase. Improved binding of the sensorimotor elements after tDCS is suggested by the reduction of the CURSOR response latency and CMC time delay after anodal tDCS, when compared to sham tDCS. This result is in agreement with our prior work (Dutta et al., 2014) that showed that offline M1 anodal tDCS decreased the delay in initiation of TA contraction when compared to sham tDCS. However, in that study, we did not find a significant effect of anodal tDCS on the CMC peak during muscle contraction in VMT, although a significant effect of anodal tDCS on the CMC time delay was present. In another study (Dutta and Chugh, 2012) with a larger sample size (*N* = 40), we found that anodal tDCS induced a statistically significant CMC enhancement of the tibialis anterior muscle during quiet standing with eyes closed, 45 and 60 min after the end of tDCS. Therefore, the effect of anodal tDCS on CMC needs further investigation with larger sample sizes.

Based on the computational and experimental results of the present study as well as earlier works (Dutta and Nitsche, 2013), we suggest that the changes to the slope of the I-O curve of ePN, e.g., an increase by anodal tDCS and a decrease by cathodal tDCS, is reflected by the TMS-MEP SR curve (Nitsche et al., 2005). Nitsche et al. (2005) suggested that the initial effects of tDCS primarily depends on subthreshold resting membrane potential changes, which are able to modulate the I-O curve, but not motor thresholds. Such change in the I-O curve was simulated by the SSLC by changing the sigmoid function that scales controller behavior (Stefanovic and Galiana, 2014a). In accordance, the results from the current study demonstrate that post-tDCS the adapted I-O sigmoid, S_post−tDCS_, increased muscle activation levels (EMG amplitude) and improved response time (correlate of CURSOR response latency) of task performance. This suggests that the muscle response frequency (i.e., the bandwidth of movement dynamics), as well as the gain of the urgency of the respective movement increase (i.e., speed and force; Thobois et al., 2007) increased post-tDCS. Our computational results are qualitatively in accordance with the experimental results showing that that anodal tDCS improved EMG response latency, CURSOR response latency as well as CMC time delay (p < 0.05). No effects of tDCS on the spinal aspects of SSLC were considered, as parametrized by interneurons (IN), Renshaw Cells (RC), Motoneurons (Mn), and Spindles (SM), as shown in Figure 2. Our adaptive SSLC approach separating the cortical effects—in the “motor urgency” sigmoidal function of the SSLC (Stefanovic and Galiana, 2014a)—from the spinal effects is novel since tDCS aftereffects are usually lumped into corticospinal excitability alterations based on neurophysiological testing by TMS. Our novel adaptive cortico-spinal-like controller was fitted to the neurophysiological test data to simulate the behavioral VMT effects. Therefore, the results of the gray-box analysis show that the cortical excitability alterations (captured with a TMS-MEP SR curve) can explain the behavioral effects of anodal tDCS during VMT (ballistic myoelectric control) without any change of spinal model parameters. Our modeling results showed an effect of anodal tDCS on the “motor urgency” (Thobois et al., 2007).

Regarding clinical relevance of the computational results, the change in the slope of the I-O curve of ePN may be able to compensate for small changes in the signal conduction speeds (e.g., due to demyelination, Wallerian degeneration) that needs further investigation. We specifically modeled co-contraction in the SSLC since Roche and colleagues showed that anodal tDCS induces effects on spinal network excitability similar to those observed during co-contraction, suggesting that anodal tDCS activates descending corticospinal projections mainly involved in co-contractions (Roche et al., 2011). In this study on healthy individuals, the signal conduction delays are assumed to be constant, and thus do not have dynamic effects on the control output. Here, tDCS-facilitated stroke rehabilitation has been of particular interest because stroke is a global health problem and fourth leading cause of disability worldwide (Strong et al., 2007; Sacco et al., 2013). Several small studies have shown beneficial effects of tDCS on motor functions that mimic activities of daily living in patients with chronic stroke and suggested that tDCS may play an adjuvant role (Hummel et al., 2005; Galea and Celnik, 2009). The most frequent stroke-related disability (39–90%) is the impairment of walking (Weerdesteyn et al., 2008), and therefore mechanisms of tDCS effects of leg motor cortex representations (M1) are relevant, but not extensively explored so far. Also, multiple sclerosis (MS) is one of the most prevalent diseases of the central nervous system (CNS) with recent prevalence estimates indicating that MS directly affects 2.3 million people worldwide (Browne et al., 2014). MS is an immune-mediated disease characterized by inflammatory demyelination and neurodegeneration within the CNS. In MS, falls continue to present as a common and serious health concern that can be as high as 56% (Hayes et al., 2017). People with MS with a history of falls report significantly poorer physical and psychological health status compared with non-fallers with MS therefore prevention of falls is critical for the quality of life with MS. Here, anodal tDCS may change the input-output (I-O) function of the primary pyramidal neurons (Lafon et al., 2017) that constitutes the descending cortical command, thereby improving muscle control. In order to understand the effects of signal conduction speeds, we can analyse the SSLC model. In SSLC, changes in the signal conduction speeds manifest as signal delays, *e*^−δ*s*^, between the cortical and spinal centers. Since the value *e*^−δ*s*^ decays as time delay increases, the effect of the cortical command decreases relative to the co-contraction level. In other words, increased signal delay times causes higher levels of spasticity, and spasticity has an association with the quality of life (QOL). After severe demyelination, conduction speeds can drastically reduce leading to significant signal delay times, where *e*^−δ*s*^ approaches zero so that only the co-contraction remains:

$$\begin{array}{l} U_{T}\left(s\right)=\frac{CU_{G}\left(s\right)}{1+M_{T}\left(s\right)}\\ U_{G}\left(s\right)=\frac{CU_{T}\left(s\right)}{1+M_{G}\left(s\right)} \end{array}$$

Now, if both sides of the controller (see Figure 2B) are driven only by the cross-connections of antagonist muscles without cortical command, the antagonist muscle activation thresholds would also be zero leaving both muscles flaccid. Our modeling showed that increased signal delay times causes higher levels of spasticity, and spasticity has an association with the quality of life. In future clinical studies, our adaptive cortico-spinal-like controller will be applied on stroke and MS subjects suffering from lower limb spastic or flaccid paralysis to dissociate tDCS effects into cortical vs. spinal effects that contribute to behavioral results during VMT with tDCS when compared to sham tDCS.

## Author contributions

VK carried out the computational modeling under the guidance of FS and AD. AF, SB, and MN substantially contributed to the neurophysiological interpretation of the computational results. FS, AD, AF, SB, and MN have substantially contributed to the scientific analysis of this work. All authors have also drafted the work and revised it critically with contribution related to author order. All authors have approved the final version before submission.

### Conflict of interest statement

MN is member of the Scientific Advisory Board of Neuroelectrics. The other authors declare that the research was conducted in the absence of any commercial or financial relationships that could be construed as a potential conflict of interest.
